# Sex disparities in papillary thyroid cancer survival: Divergent patterns of relative and absolute effects across the age spectrum

**DOI:** 10.1371/journal.pone.0328876

**Published:** 2025-07-24

**Authors:** Hui Ouyang, Xiaolin Dou, Xinying Li, Mingyu Cao, Zhijing Wu, Fada Xia

**Affiliations:** 1 Department of General Surgery, Xiangya Hospital, Central South University, Changsha, Hunan Province, China; 2 National Clinical Research Center for Geriatric Disorders, Xiangya Hospital, Central South University, Changsha, Hunan Province, China; Washington University in St Louis, UNITED STATES OF AMERICA

## Abstract

**Purpose:**

This retrospective cohort study aimed to reevaluate the prognostic impact of sex and determine whether age modifies the effect of sex on cancer-specific survival (CSS) in PTC patients.

**Methods:**

Data for PTC patients diagnosed between 2004 and 2015 were retrieved from the SEER database. The primary outcome was CSS. The effect of Sex was evaluated using both relative (hazard ratios, HRs) and absolute measures (survival differences). Additionally, the effect of sex modified by age was assessed using restricted cubic spline curves from the Cox and Poisson models, with further analysis of the interaction between sex and age.

**Results:**

Of 77,349 patients, 16,152 (20.9%) were male. Men exhibited older age, more aggressive clinicopathological features, and received more radioactive iodine treatment. Multivariate Cox analysis determined male sex as an independent risk factor (adjusted HR: 1.46 (1.24–1.70). The 10-year and 15-year survival differences between men and women were 0.46% (95% CI, 0.25%−0.67%) and 0.77% (95% CI, 0.31%−1.22%), respectively. Moreover, a nonlinear effect for sex across age was observed, with HRs for men plateauing below age 50 and decreasing thereafter. Importantly and conversely, before age 50, the absolute survival difference increased slightly with age, but after 50, it significantly widened. Furthermore, A significant negative multiplicative interaction between sex and age was found.

**Conclusions:**

Our analyses provide robust evidence that male sex is indeed an independent risk factor for CSS in PTC patients. Although younger female patients show a relative survival advantage, this does not translate into a substantial absolute benefit, which widens with advancing age.

## Introduction

Thyroid cancer has emerged as one of the fastest-growing cancer diagnoses worldwide, with its incidence surging by an alarming 313% over the past four decades [1]. Among the various types of thyroid malignancies, papillary thyroid cancer (PTC) is the most prevalent, accounting for 80–90% of all thyroid cancer cases. Notably, there is a significant gender disparity in both the incidence and initial presentation of PTC. Specifically, women are diagnosed with PTC at a rate three to four times higher than that of men [2]. Conversely, men tend to present with more advanced disease and at an older age at diagnosis. These gender-specific differences have sparked considerable debate regarding the prognostic implications of male gender in PTC.

To date, there has been little consensus on the impact of sex on PTC cancer specific survival (CSS). Although univariate analyses in numerous research have consistently shown that male sex is associated with poorer CSS. The results of multivariate analyses have yielded inconsistent findings across different studies [3–6]. A prospective multicenter study involving 3,572 PTC patients by Jonklaas et al. [6] reported a significant crude hazard ratio (HR) of 0.40 for CSS for women, but this association lost significance in multivariate analysis with an adjusted HR of 0.72. Further stratified analysis by age group in this study indicated women before age 55 had better CSS than men, but this advantage disappeared in elder age groups. Similarly, research by Cintron-Garcia et al reported no survival difference between men and women in PTC patients older than 55 years [7]. These findings suggest that the relationship between sex and CSS in PTC may be limited by a small sample size and modified by age. Despite these insights, the existing literature primarily focuses on the relative effects (hazard ratios) of sex within different age subgroups, without addressing the dynamic changes in the effect of sex modified by age or exploring the absolute survival differences between sexes across the age spectrum.

Given the conflicting results on Sex impact on CSS and limited research investigating the interaction between sex and age on CSS in PTC, this study aimed to reevaluate the prognostic impact of sex, and determine whether age modifies the effect of sex on CSS in PTC patients. We analyzed data from the Surveillance, Epidemiology, and End Results (SEER) database to investigate the difference in CSS between sexes, assess the dynamic influence of sex across the age spectrum, and explore the interaction effect between sex and age.

## Materials and methods

This study employed a retrospective cohort design, using data from the SEER database for PTC patients diagnosed between 2004 and 2015. Comparing to the newer version (2000–2020 dataset), the 2004–2015 dataset provides a longer follow-up time and uniform variable coding, such as “CS tumor size (2004-2015)”, “CS extension (2004-2015)”, “CS lymph nodes (2004-2015)”, and “CS mets at dx (2004-2015)”. These variables are crucial for deriving the 8th edition of the AJCC TNM staging. The primary outcome was CSS, and the effect of sex was evaluated using both relative and absolute measures. All analysis scripts are available at https://github.com/HUI950319/PTC-SEER-SurvivalStudy-Sex_Age.

### Ethical statements

This study utilized data obtained from the Surveillance, Epidemiology, and End Results (SEER) database, a publicly accessible resource that provides de-identified patient information. As such, this research did not involve any direct interaction with human participants or animals, nor did it entail clinical trials. Consequently, the study is exempt from the requirement for ethical review and approval in accordance with institutional guidelines and the Declaration of Helsinki. The SEER database ensures the confidentiality and anonymity of the data, complying with relevant ethical standards for public health research.

### Patients and data collection

Data for patients diagnosed with papillary thyroid cancer (PTC) (ICD-O-3 codes: 8050, 8260, 8340–8344, 8350, 8450–8460) were retrieved from the Surveillance, Epidemiology, and End Results (SEER) database (Incidence – SEER Research Plus Data, 17 Registries, November 2021 Sub (2000–2019)). This study focused on a subset of PTC patients diagnosed between 2004 and 2015. Inclusion criteria consisted of PTC as the first primary cancer, diagnosis confirmed through histological or cytological pathology, and histological grade of well, moderately, or poorly differentiated. Exclusion criteria included unknown demographic, clinicopathological, or follow-up information, as well as follow-up times of less than one month. Finally, a total of 77,349 PTC patients were included in this study (S1 Fig in S1 File). Given the public availability of SEER data, this study was exempt from approval by the Institutional Review Board of Xiangya Hospital.

### Variables

Collected variables included demographic characteristics: age at diagnosis, sex, and race; clinicopathologic characteristics: grade, tumor size, extrathyroidal extension, multifocality, nodal metastasis site, regional nodes examined, regional nodes positive, and distant metastasis; therapy information: surgery, radiotherapy, and chemotherapy; and follow-up data: survival months and cause of death. Additionally, the 8th edition of the AJCC TNM stage was used and computed based on the tumor characteristics, following the guidelines of the 8th edition of the AJCC staging manual. The primary outcome for this study was cancer-specific survival (CSS), calculated from the initial diagnosis to either death from thyroid cancer or the last follow-up, whichever occurred first.

### Statistical analysis

Survival curves were used to characterize the survival probability between different sex groups. The unadjusted survival curves were generated based on the Kaplan-Meier method. While adjusted survival curves utilized a direct method based on multivariate Cox model [8,9]. The effect of sex on CSS was evaluated using both relative (hazard ratios, HRs) and absolute measures (absolute survival differences). HRs with 95% confidence intervals (CIs) were estimated using Cox models. While absolute survival differences were calculated based on the survival curves, and visualized with risk-difference curves [10]. In multivariable models, adjustments were made for the following covariates: age, race, tumor grade, size, extension, multifocality, M stage, and treatment modalities (surgery, radiotherapy, chemotherapy), as well as the interaction term between age and sex.

To explore whether the effect of sex on CSS is dependent on age, HRs for sex across the age spectrum as a continuous variable were obtained from the three-knot restricted cubic spline (RCS) Cox model [11]. Additionally, incidence rates (person-years death rate) for sex across age were estimated using a three-knot RCS Poisson model [12] with an offset of 1000 person-years. The knots for the RCS models were strategically placed at the 10th, 50th, and 90th percentiles of the age distribution. The nonlinearity of the association in RCS models was assessed using a Wald test [13].

To investigate the potential biological mechanisms between sex and age, the interaction effect analysis was further explored. Age was categorized into two groups: less than 50 years and 50 years or older. The effect of different sex and age groups on CSS was also assessed with HRs and absolute survival differences. Additionally, interactions between sex and age were evaluated on both multiplicative and additive scales [14,15]. Additive interaction suggests that the combined effect of two variables is greater (or smaller) than the sum of the individual effects of the two exposures. Conversely, interaction on a multiplicative scale indicates that the combined effect is larger (or lesser) than the product of the individual effects. Additive interaction was evaluated using three measures: relative excess risk due to interaction (RERI), attributable proportion due to interaction (AP), and synergy index (SI). No additive interaction was deﬁned as a 95%CI of RERI and AP including 0, and a 95% CI of SI comprising 1. The 95% CIs for RERI, AP, and SI were calculated using the delta method [16].

To test the robustness of our results, several sensitivity analyses were conducted. Firstly, survival and interaction analyses were repeated using the Fine and Gray competing risk model, considering non-thyroid cancer death as a competing risk. Secondly, the E-value measure was employed to assess the potential impact of unmeasured confounding factors on the overall effect of sex on CSS [17]. Thirdly, the effect of sex on CSS was estimated stratified by various age subgroups. Finally, interaction analyses were repeated with an age cutoff of 50 years.

Summary statistics were presented as total frequencies and percentages for categorical variables, while median values with interquartile ranges or means and standard deviations (SD) were reported for continuous variables, as appropriate. Differences in data distribution between groups for both categorical and continuous variables were assessed by the χ2 test and Mann-Whitney U test, respectively. R version 4.3.1 was used to perform all statistical analyses and create all figures.

## Results

### Patient characteristics

A total of 77,349 PTC patients were identified from the SEER database for the period spanning 2004–2015 (S1 Fig in S1 File). The patient characteristics are summarized in Table 1. Of the total cohort,

**Table 1:** Characteristics of patients with papillary thyroid cancer according to sex.

Variable	Overall (N = 77,349)	Female (N = 61,197)	Male (N = 16,152)	p-value
**Age (years)**	48.00 (37.00, 58.00)	47.00 (37.00, 57.00)	51.00 (41.00, 61.00)	<0.001
**Race**				<0.001
White	63,825 (82.5%)	50,041 (81.8%)	13,784 (85.3%)	
Black	4,621 (6.0%)	3,927 (6.4%)	694 (4.3%)	
Other	8,903 (11.5%)	7,229 (11.8%)	1,674 (10.4%)	
**Grade**				<0.001
Well differentiated	13,624 (17.6%)	10,585 (17.3%)	3,039 (18.8%)	
Moderately differentiated	2,584 (3.3%)	2,018 (3.3%)	566 (3.5%)	
Poorly differentiated	419 (0.5%)	279 (0.5%)	140 (0.9%)	
Unknown	60,722 (78.5%)	48,315 (78.9%)	12,407 (76.8%)	
**Tumor_size (mm)**	12.00 (6.00, 23.00)	12.00 (6.00, 21.00)	15.00 (8.00, 30.00)	<0.001
**Extension** ^a^				<0.001
No	65,151 (84.2%)	52,218 (85.3%)	12,933 (80.1%)	
3b	10,163 (13.1%)	7,549 (12.3%)	2,614 (16.2%)	
4a	1,496 (1.9%)	1,037 (1.7%)	459 (2.8%)	
4b	539 (0.7%)	393 (0.6%)	146 (0.9%)	
**Multifocality**	32,451 (42.0%)	24,964 (40.8%)	7,487 (46.4%)	<0.001
**Number of examined lymph nodes**	1.00 (0.00, 3.00)	1.00 (0.00, 3.00)	1.00 (0.00, 5.00)	<0.001
**Number of positive lymph nodes**	13.00 (0.00, 98.00)	12.00 (0.00, 98.00)	15.00 (1.00, 98.00)	<0.001
**T stage**				<0.001
T1	49,419 (63.9%)	40,675 (66.5%)	8,744 (54.1%)	
T2	12,195 (15.8%)	9,232 (15.1%)	2,963 (18.3%)	
T3a	3,537 (4.6%)	2,311 (3.8%)	1,226 (7.6%)	
T3b	10,163 (13.1%)	7,549 (12.3%)	2,614 (16.2%)	
T4a	1,496 (1.9%)	1,037 (1.7%)	459 (2.8%)	
T4b	539 (0.7%)	393 (0.6%)	146 (0.9%)	
**N stage**				<0.001
N0/Nx	61,216 (79.1%)	49,780 (81.3%)	11,436 (70.8%)	
N1a	11,171 (14.4%)	8,209 (13.4%)	2,962 (18.3%)	
N1b	4,962 (6.4%)	3,208 (5.2%)	1,754 (10.9%)	
**M stage**				<0.001
M0	76,890 (99.4%)	60,937 (99.6%)	15,953 (98.8%)	
M1	459 (0.6%)	260 (0.4%)	199 (1.2%)	
**TNM stage**				<0.001
I	69,781 (90.2%)	56,218 (91.9%)	13,563 (84.0%)	
II	6,447 (8.3%)	4,266 (7.0%)	2,181 (13.5%)	
III	641 (0.8%)	418 (0.7%)	223 (1.4%)	
IVA	245 (0.3%)	174 (0.3%)	71 (0.4%)	
IVB	235 (0.3%)	121 (0.2%)	114 (0.7%)	
**Surgery**				0.736
Lobectomy	9,974 (12.9%)	7,904 (12.9%)	2,070 (12.8%)	
Total thyroidectomy	67,375 (87.1%)	53,293 (87.1%)	14,082 (87.2%)	
**Radiotherapy**				<0.001
None/Unknown	39,511 (51.1%)	32,197 (52.6%)	7,314 (45.3%)	
Radioisotopes	37,838 (48.9%)	29,000 (47.4%)	8,838 (54.7%)	
**Chemotherapy**				0.085
No/Unknown	77,232 (99.8%)	61,112 (99.9%)	16,120 (99.8%)	
Yes	117 (0.2%)	85 (0.1%)	32 (0.2%)	
**Follow-up (months)**	101.00 (69.00, 137.00)	101.00 (70.00, 138.00)	97.50 (68.00, 134.00)	<0.001
**Cancer-specific mortality**				<0.001
Survival	72,598 (93.9%)	58,057 (94.9%)	14,541 (90.0%)	
Death of PTC	833 (1.1%)	474 (0.8%)	359 (2.2%)	
Death of other causes	3,918 (5.1%)	2,666 (4.4%)	1,252 (7.8%)	

Abbreviations: AJCC, American Joint Committee on Cancer.

^a^categories 3b, 4a, and 4b in extension according to T stages 3b, 4a, and 4b.

61,197 patients (79.1%) were female, with a median follow-up duration of 101 months, while 16,152 patients (20.9%) were male, with a median follow-up of 97 months. At presentation, men tended to be slightly older than women (median age, 51 vs. 47 years, p < 0.001), with a higher proportion of white race, and moderately or poorly differentiated histology. Moreover, men had significantly more advanced disease at presentation, including larger primary tumor size, a higher proportion of aggressive extension, a higher proportion of multifocality, a greater number of positive lymph nodes, as well as more advanced T, N, M, TNM stage (all p < 0.001). Notably, radioactive iodine (RAI) was administered more frequently to men than to women (54.7% vs. 47.4%, p < 0.001), but no significant differences were found in surgery and chemotherapy between sexes.

### Relative and absolute effect of sex on CSS

**Fig 1:**
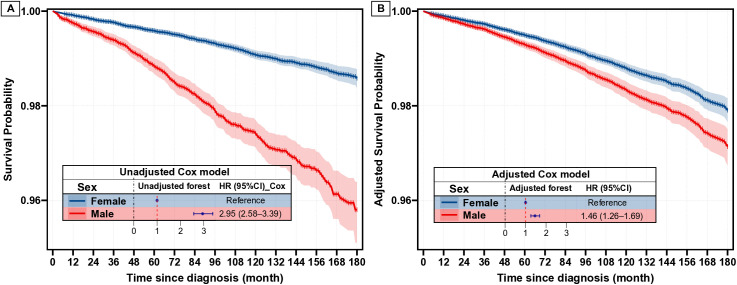
Survival curves for female and male based on unadjusted (A) and adjusted (B) Cox model.

**Fig 2:**
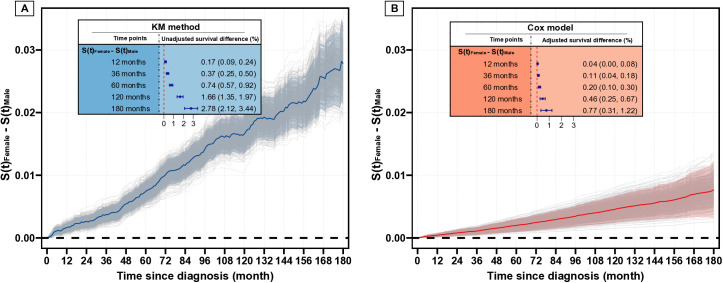
Survival difference curves between female and male based on unadjusted (A) and adjusted (B) Cox model.

In the Kaplan-Meier analysis (Fig 1A), cancer-specific survival (CSS) declined sharply for men, while the decline for women was more modest (unadjusted HR for men vs. women, 2.95, 95% CI, 2.58–3.39). After adjusting for covariates in multivariate analysis (Fig 1B), although the decline in CSS for men was less steep than that observed in the Kaplan-Meier curve, men still had a worse CSS compared to women, with an adjusted HR of 1.46 (95% CI, 1.26–1.69). Furthermore, we investigated the absolute survival differences between sexes. Men consistently presented a significantly unadjusted survival difference compared to women over time (Fig 2A). This finding was in line with the adjusted curves (Fig 2B). Specifically, the unadjusted 10-year and 15-year survival differences between men and women were 1.66% (95% CI, 1.35%−1.97%) and 2.78% (95% CI, 2.12%−3.44%), respectively. After adjustment, although the survival differences remained significant, the 10-year and 15-year survival differences between men and women were reduced to 0.46% (95% CI, 0.25%−0.67%) and 0.77% (95% CI, 0.31%−1.22%), respectively. Notably, similar results were observed in the competing risk analysis (S2 and S3 Figs in S1 File). Besides, a relatively large E-value of 2.28 was observed for the adjusted HR of sex, further confirming the robustness of the finding that sex is an independent factor influencing CSS in PTC patients.

### Effect of sex across age spectrum

**Fig 3:**
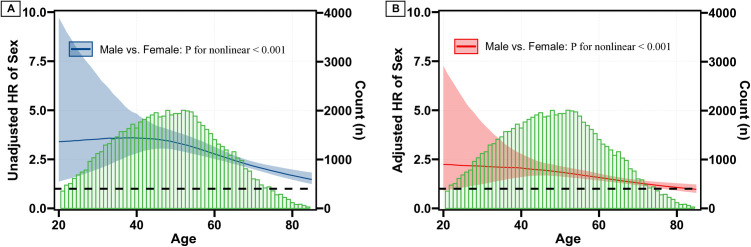
Hazard ratios for Sex across age based on unadjusted (A) and adjusted (B) Cox model. Note: shaded areas representing the 95% confidence intervals; green bars indicating the distribution of age among all patients.

**Fig 4:**
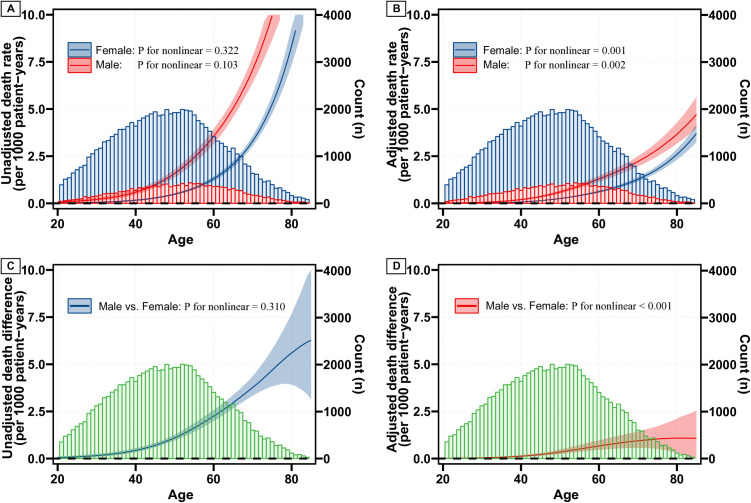
Death rate and difference for Sex across Age without (A, C) or with (B, D) adjustment. Note: shaded areas representing the 95% confidence intervals; blue and red bars indicating the distribution of age for females and males, respectively; green bars indicating the distribution of age among all patients.

As shown in the restricted cubic spline (RCS) curves based on the Cox model (Fig 3), the unadjusted HRs for men vs. women remained at a high plateau (HR of 3.5) for individuals below the age of 50, and subsequently decreased with increasing age, with the survival advantage of women diminishing around the age of 80 (Fig 3A). This trend was also observed in the adjusted HRs for sex (Fig 3B). Of note, a nonlinear trend for the HRs of sex was observed (p < 0.001). Furthermore, in the RCS curves based on the Poisson model (Fig 4), the unadjusted death rate (per 1,000 person-years) increased with age for both sexes, with a more rapid increase in men than in women (Fig 4A). Consequently, the unadjusted death difference per 1,000 person-years between men and women also escalated with age (Fig 4C). A similar trend, albeit with reduced differences, was observed in the adjusted death rate (Fig 4B) and the death difference between sexes (Fig 4D). Notably, the differences were very small when age was below 50, and a nonlinear trend was observed in the adjusted absolute death difference by sex (Fig 4B,D).

To assess the robustness of the dynamic effect of sex across the age spectrum, we further examined the relative and absolute effects of sex stratified by different age groups. Overall, the results for HRs (S4 and S5 Figs in S1 File) and 10-year survival probabilities (S6 and S7 Figs in S1 File) of sex across age groups were consistent with the dynamic effect of sex observed in Fig 4.

### Interaction effect between sex and age

**Fig 5:**
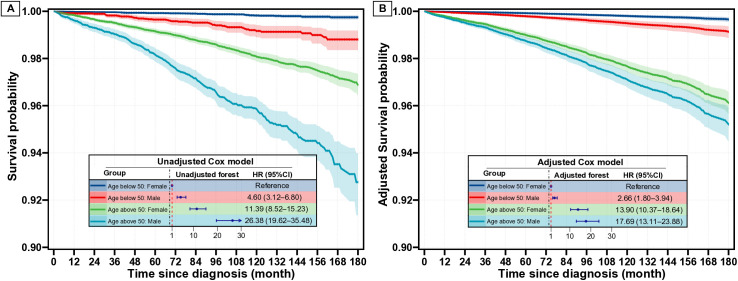
Survival curves for different sex and age groups based on unadjusted (A) and adjusted (B) Cox model.

To further explore the interaction effect between sex and age, we stratified the patients into four groups: women below 50, men below 50, women above 50, and men above 50 based on the changepoint at age 50 as shown in Fig 3A and 3B. The survival curves derived from the Cox model (Fig 5) demonstrated a more pronounced survival difference across age groups compared to sex groups, with adjusted HRs (95%CI) for men below 50, women above 50, and men above 50 compared to women below 50 as 2.66 (1.80–3.94), 13.90 (10.37–18.64) and 17.69 (13.11–23.88), respectively. Notably, the 10-year survival probabilities for women and men were both above 99% when age were below 50 (Fig 4 and S6 Fig in S1 File). Additionally, the absolute survival difference between women and men is relatively greater in age above 50 than that in age below 50 (detailed in S8 and S9 Figs in S1 File). The results from the Cox analysis were further supported by the competing risk analysis (S7, S10–S12 Figs in S1 File).

In the analysis of interaction effects (Table 2), we found that the multiplicative scale for the interaction between sex and age was 0.48 [0.31, 0.73] in the adjusted Cox model. This finding indicates a negative multiplicative interaction between sex and age, suggesting that the effect of sex on the CSS is diminished by the increase in age. Furthermore, both the RERI and AP included 0 (RERI, 2.13 [−0.50, 4.77] AP, 0.12 [−0.02, 0.26]), whereas the SI contained 1 (SI, 1.15 [0.97, 1.35] (Table 2). These findings suggest no evidence of an additive interaction between sex and age. The interaction analysis using the competing risk model also indicated a significant negative multiplicative interaction between sex and age (S1 Table in S1 File). In the sensitivity analysis based on an age cutoff of 55 (S2 and S3 Tables in S1 File), a significant negative multiplicative interaction effect between sex and age was also identified, again with no evidence of a significant additive interaction.

**Table 2:** Interaction analysis of sex and age in the adjusted Cox model based on age cutoff of 50.

	Female sex	Male sex	Effect of Sex within the strata of Age
	HR [95% CI]	HR [95% CI]	HR [95% CI]
Age below 50	1.00 [Reference]	2.66 [1.80, 3.94]	2.66 [1.80, 3.94]
Age above 50	13.9 [10.37, 18.64]	17.69 [13.11, 23.88]	1.27 [1.09, 1.49]
Effect of Age within the strata of Sex	13.9 [10.37, 18.64]	6.65 [4.92, 8.99]	
Multiplicative scale	0.48 [0.31, 0.73]		
RERI	2.13 [−0.50, 4.77]		
AP	0.12 [−0.02, 0.26]		
SI	1.15 [0.97, 1.35]		

Abbreviations: RERI, relative excess risk due to interaction; AP, attributable proportion due to interaction; SI, synergy index.

## Discussion

PTC exhibits remarkable gender differences in both incidence and initial presentation. However, the impact of sex on CSS in PTC remains a topic of ongoing debate, and there is limited research regarding the dynamic influence of sex across the age spectrum, and interaction analysis between sex and age. Our large-scale population-based study demonstrated that men have significantly poorer CSS compared to women in all PTC patients. This finding is robustly supported by both relative (hazard ratios, HRs) and absolute (survival difference) measures, as evidenced by the multivariate Cox model and the Fine-Gray model. Additionally, we found that the HRs for men vs. women had a high plateau for individuals below the age of 50, followed by a gradual decrease with increasing age, with the survival advantage of women diminishing around the age of 80. Importantly, before the age of 50, the death difference (death rate per 1,000 person-years between sexes) increased slightly with age. However, after the age of 50, this difference significantly widened as age further increased. Furthermore, our analysis revealed a negative multiplicative interaction effect between sex and age, highlighting the complex interplay between these two critical factors in determining PTC outcomes.

The present study is consistent with other studies showing that men tend to present with more advanced disease, which contributes to a poor CSS in KM analysis or univariate analysis [18–20]. Notably, we demonstrated that men still exhibit a poor prognosis in multivariate analysis, while several studies reported that sex was not an independent factor for CSS in PTC [3–5,21,22]. The inconsistent results from these studies might be attributed to relatively smaller cohorts [5,21,22], and complex interactions between sex and histology [3,4,21] or age [3,5,21,22]. This is exemplified by the study by Hollenbeak et al., which reported that males with stage I PTC had a minimally reduced CSS, and stage-stratified analysis revealed no CSS difference between male and female patients with follicular thyroid cancer (FTC). Our analysis exclusively includes PTC patients, which aligns with recent studies highlighting that male gender was an independent risk factor for CSS in PTC [18,23,24]. Collectively, our findings robustly demonstrate that sex itself is an independent factor for CSS in PTC, providing valuable insights into the prognostic implications of gender in this thyroid cancer subtype of PTC. However, it is crucial to acknowledge that the impact of sex on survival outcomes may vary across different thyroid cancer histologies. Further research is warranted to elucidate the specific role of sex in other thyroid cancer subtypes.

Our study specifically focuses on assessing the dynamic influence of sex on CSS across different age groups. This involves using restricted cubic spline models to explore how the effect of sex changes with age, which is a novel aspect not fully addressed in the referenced study. One particularly interesting finding is that the HRs for men versus women demonstrated a significantly high plateau in younger age groups, indicating a consistently elevated risk for men. The observed sex disparities in PTC survival may be influenced by hormonal factors, particularly estrogen. Estrogen is known to promote thyroid cell proliferation and is more prevalent in women, which could explain the higher incidence of PTC in females. The relative survival advantage in younger women may be due to estrogen’s protective effects, which diminish with age as estrogen levels decline post-menopause. This result aligns with a recent review suggesting that younger female PTC patients may have a survival advantage due to the protective role of estrogen [25]. In contrast to the potential protective effects of estrogen, ERα was proved to to promote cell proliferation in PTC organoids [26] and was increased in post-menopausal patients [27], this might be in part explain the diminished advantage in older female patients. As age increased beyond 50, we noted a gradual decrease in HRs, suggesting a narrowing gap between men and women. This trend supports the hypothesis that the gradual reduction in estrogen levels post-menopause may explain the diminishing survival advantage for women as age increases. Notably, our analysis revealed a striking phenomenon: the survival advantage for women appeared to diminish around the age of 80, with the HRs approaching 1 at this age (Fig 3A,B). This observation provides crucial insights into the age-dependent nature of sex-based differences in PTC outcomes. The age-dependent pattern of sex-based HRs offers a compelling explanation for the seemingly contradictory findings in previous studies. It elucidates why some investigations reported an insignificant overall effect of sex on survival, while significant impacts were found when stratifying by age [4,6,7].

Another important finding of our study is that the absolute survival difference between the sexes exhibits a distinct pattern compared to the trends observed in HRs across age groups. Before the age of 50, the difference in death rate per 1,000 person-years increased slightly, with the 10-year survival probability for both women and men remaining above 99%. This gradual widening and small mortality gap might be related to the small baseline risk for death in younger patients. As a result, despite relatively high HRs between men and women at a young age, the absolute survival difference in this age group remains minor. Perhaps the most striking finding emerged in patients over 50, where we observed a significant and progressive widening of the mortality gap as age increased. This suggests that while the relative risk (HRs) may decrease, the absolute impact of sex on survival becomes increasingly substantial in older age groups. This finding aligns with a recent study that reported the HRs for disease-specific survival (DSS) becoming similar for men and women after the age of 55, although it did not address the increased 15-year survival difference [6]. Likewise, this phenomenon can be explained by the increasing baseline risk of death with advancing age, likely resulting from the cumulative effect of risk factors and the potential for more aggressive tumor progression in men as age progresses. Together, these findings underscore the importance of considering absolute measures (survival difference) when assessing prognostic factors, particularly in younger PTC patients, where the baseline mortality rate is low and the 10-year survival rate exceeds 99%.

We also investigate the interaction effect between sex and age, which is crucial for understanding how these factors interplay to affect CSS in PTC patients. Our analysis uncovered a significant negative multiplicative interaction effect between sex and age, shedding new light on the complex interplay between sex and age in determining PTC outcomes. The negative interaction suggests that the impact of sex on PTC survival outcomes is not constant across different age groups but rather diminishes as age increases. This pattern further confirms our observations of decreasing HRs for men versus women in older age groups. the underlying biological mechanisms for negative interaction between sex and age might be explained by hormonal influences. As estrogen levels decline with age, particularly post-menopause, the protective effect in women may diminish, potentially contributing to the observed interaction. This interaction analysis provides insights into how the impact of sex on survival outcomes varies across different age groups, adding depth to the understanding of sex disparities in PTC.

Several limitations need to be noted regarding the present study. Firstly, although the SEER database offers a large, population-based sample with long-term follow-up, its retrospective nature may introduce inherent biases. Secondly, our analysis did not account for various histological subtypes and PTC variants. These distinct subtypes and variants could potentially act as confounding factors or effect modifiers, potentially influencing the observed associations and outcomes [3,4,7]. Future work is planned to stratify analyses by these important histological distinctions. Thirdly, the absence of recurrence data in the SEER database precludes a comprehensive assessment of sex-specific impacts on recurrence risk, necessitating further investigation in this area. Furthermore, the SEER database lacks information on several potentially sex-specific confounding factors. For instance, the presence of chronic lymphocytic thyroiditis [28] and BRAF V600E [29], which have been shown to exhibit sex-based differences, could serve as potential confounders.

## Conclusion

In conclusion, our large population-level analyses provide robust evidence that male sex is indeed an independent factor exerting a significant influence on cancer-specific survival (CSS) in patients with papillary thyroid cancer (PTC). Importantly, the age-dependent sex-based effect manifests differently in relative risk and survival differences. Although a significant survival advantage based on hazard ratios (HRs) is observed in younger female patients, this relative risk disparity in sex does not translate into a substantial absolute survival benefit. Contrary to the gradually decreasing HRs pattern in older age groups, the absolute survival difference between sexes widens significantly. This divergence underscores the increasingly substantive role of sex in determining survival outcomes among older PTC patients.

## Supporting information

S1 File S1 Text. R codes. S1 Table. Interaction analysis of sex and age in the adjusted Fine-Gray model based on age cutoff of 50. S2 Table. Interaction analysis of sex and age in the adjusted Cox model based on age cutoff of 55. S3 Table. Interaction analysis of sex and age in the adjusted Fine-Gray model based on age cutoff of 55. S1 Fig. Flowchart of patents selection. S2 Fig. Survival curves for Sex in Fine-Gray model. S3 Fig. Death difference curves between sexes in Fine-Gray model without (A) or with (D) adjustment. S4 Fig. subgroup analysis of hazard ratios between sexes across different age groups with cox model. S5 Fig. subgroup analysis of hazard ratios between sexes across different age groups with Fine-Gray model. S6 Fig. subgroup analysis of 10-years survival difference in Sex across different age groups with cox model. S7 Fig. subgroup analysis of 10-years survival difference in Sex across different age groups with fine-gray model. S8 Fig. survival difference curves between sex and age group without (A, C) or with (B, D) adjustment. S9 Fig. forest plot for survival difference between sex and age group in cox model. S10 Fig. Survival curves for sex and age groups in Fine-Gray model. S11 Fig. death difference curves between age and sex groups without (A, C) or with (B, D) adjustment. S12 Fig. forest plot for survival difference between age and sex groups in Fine-Gray model.
(ZIP)
