# Supplementary figures and images for: Sex disparities in papillary thyroid cancer survival: Divergent patterns of relative and absolute effects across the age spectrum
Source: PLoS One. 2025 Jul 24;20(7):e0328876. doi: 10.1371/journal.pone.0328876 (PMC12288988; doi:10.1371/journal.pone.0328876)

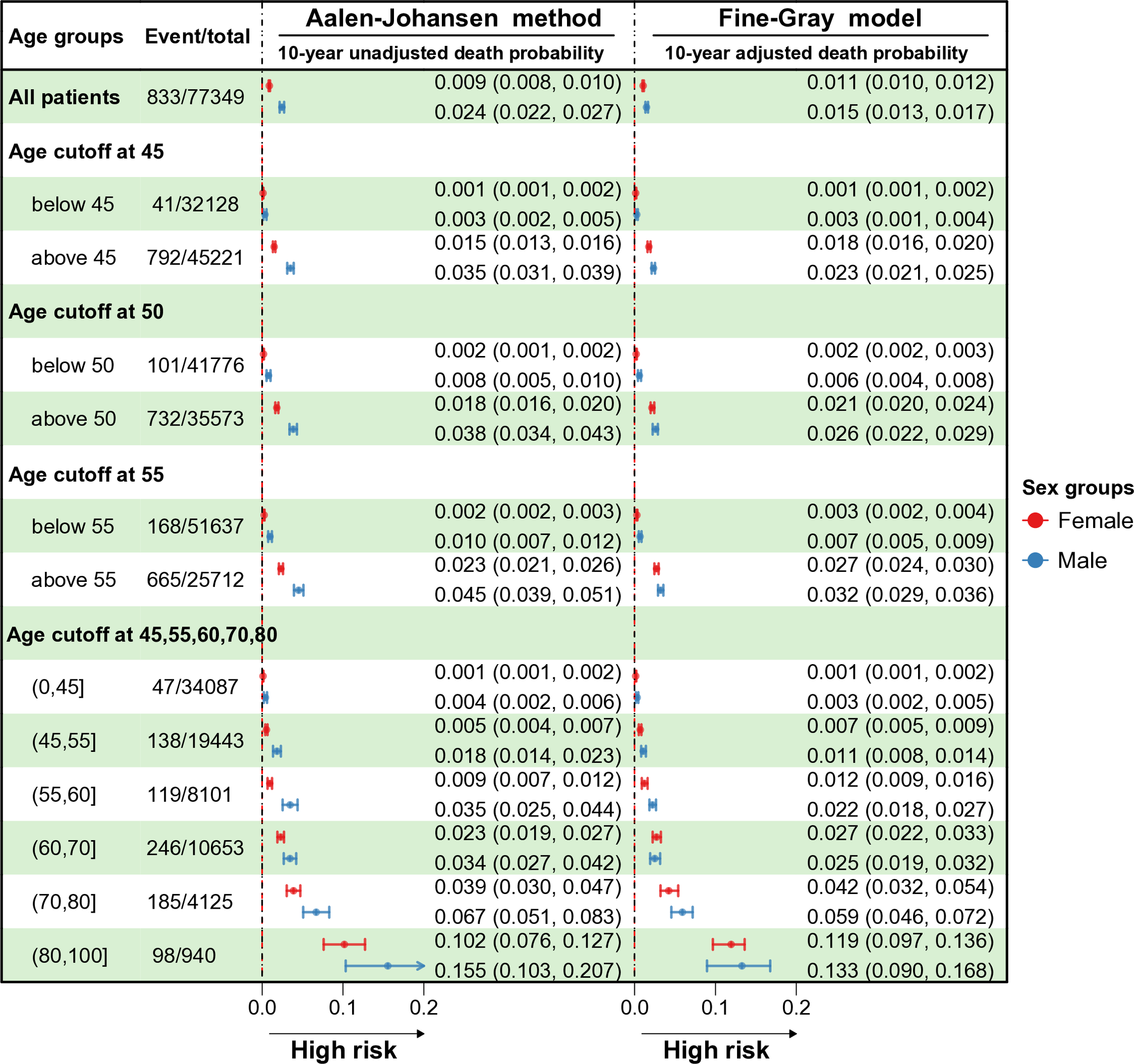

Supplement: S1 File — S1 Text. R codes. S1 Table. Interaction analysis of sex and age in the adjusted Fine-Gray model based on age cutoff of 50. S2 Table. Interaction analysis of sex and age in the adjusted Cox model based on age cutoff of 55. S3 Table. Interaction analysis of sex and age in the adjusted Fine-Gray model based on age cutoff of 55. S1 Fig. Flowchart of patents selection. S2 Fig. Survival curves for Sex in Fine-Gray model. S3 Fig. Death difference curves between sexes in Fine-Gray model without (A) or with (D) adjustment. S4 Fig. subgroup analysis of hazard ratios between sexes across different age groups with cox model. S5 Fig. subgroup analysis of hazard ratios between sexes across different age groups with Fine-Gray model. S6 Fig. subgroup analysis of 10-years survival difference in Sex across different age groups with cox model. S7 Fig. subgroup analysis of 10-years survival difference in Sex across different age groups with fine-gray model. S8 Fig. survival difference curves between sex and age group without (A, C) or with (B, D) adjustment. S9 Fig. forest plot for survival difference between sex and age group in cox model. S10 Fig. Survival curves for sex and age groups in Fine-Gray model. S11 Fig. death difference curves between age and sex groups without (A, C) or with (B, D) adjustment. S12 Fig. forest plot for survival difference between age and sex groups in Fine-Gray model. (ZIP) [file pone.0328876.s001.zip › supplementary materials/S7 Figure.tif]

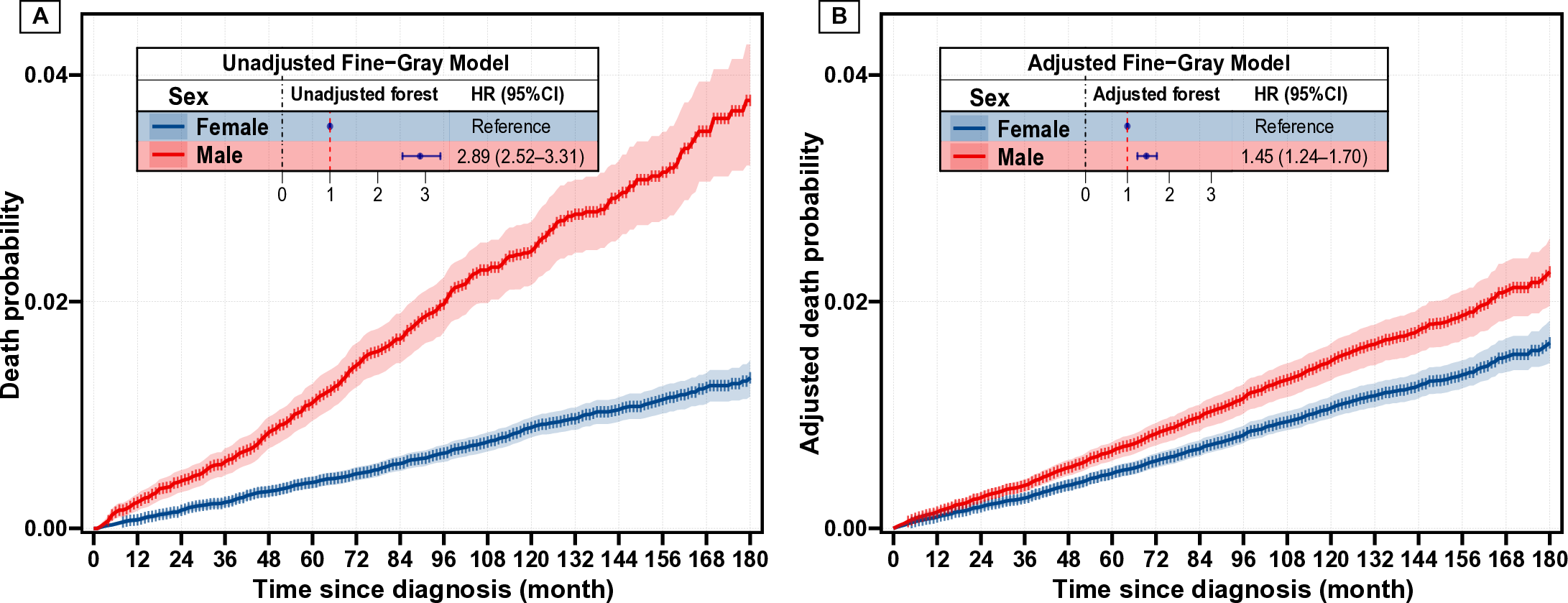

Supplement: S1 File — S1 Text. R codes. S1 Table. Interaction analysis of sex and age in the adjusted Fine-Gray model based on age cutoff of 50. S2 Table. Interaction analysis of sex and age in the adjusted Cox model based on age cutoff of 55. S3 Table. Interaction analysis of sex and age in the adjusted Fine-Gray model based on age cutoff of 55. S1 Fig. Flowchart of patents selection. S2 Fig. Survival curves for Sex in Fine-Gray model. S3 Fig. Death difference curves between sexes in Fine-Gray model without (A) or with (D) adjustment. S4 Fig. subgroup analysis of hazard ratios between sexes across different age groups with cox model. S5 Fig. subgroup analysis of hazard ratios between sexes across different age groups with Fine-Gray model. S6 Fig. subgroup analysis of 10-years survival difference in Sex across different age groups with cox model. S7 Fig. subgroup analysis of 10-years survival difference in Sex across different age groups with fine-gray model. S8 Fig. survival difference curves between sex and age group without (A, C) or with (B, D) adjustment. S9 Fig. forest plot for survival difference between sex and age group in cox model. S10 Fig. Survival curves for sex and age groups in Fine-Gray model. S11 Fig. death difference curves between age and sex groups without (A, C) or with (B, D) adjustment. S12 Fig. forest plot for survival difference between age and sex groups in Fine-Gray model. (ZIP) [file pone.0328876.s001.zip › supplementary materials/S2 Figure.tif]

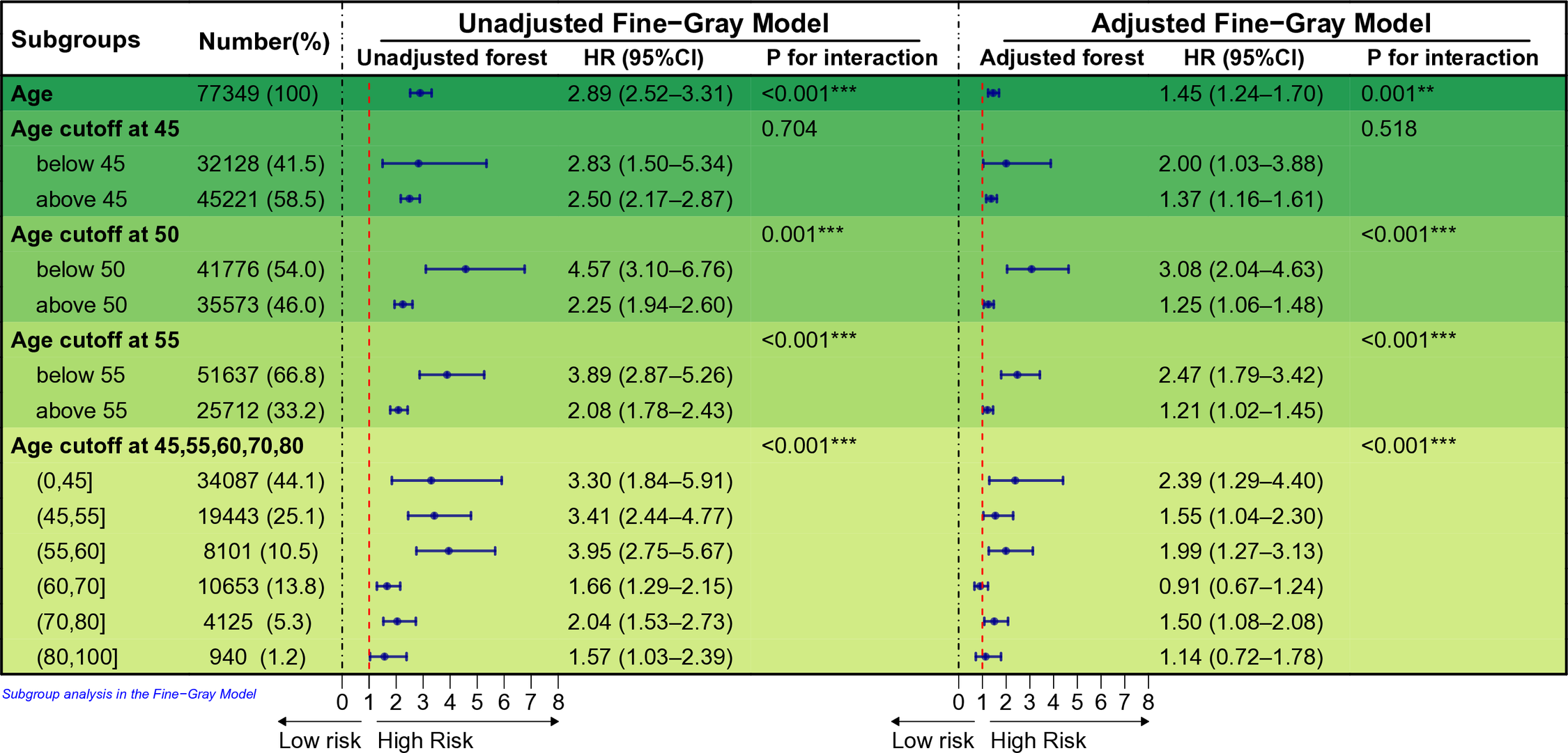

Supplement: S1 File — S1 Text. R codes. S1 Table. Interaction analysis of sex and age in the adjusted Fine-Gray model based on age cutoff of 50. S2 Table. Interaction analysis of sex and age in the adjusted Cox model based on age cutoff of 55. S3 Table. Interaction analysis of sex and age in the adjusted Fine-Gray model based on age cutoff of 55. S1 Fig. Flowchart of patents selection. S2 Fig. Survival curves for Sex in Fine-Gray model. S3 Fig. Death difference curves between sexes in Fine-Gray model without (A) or with (D) adjustment. S4 Fig. subgroup analysis of hazard ratios between sexes across different age groups with cox model. S5 Fig. subgroup analysis of hazard ratios between sexes across different age groups with Fine-Gray model. S6 Fig. subgroup analysis of 10-years survival difference in Sex across different age groups with cox model. S7 Fig. subgroup analysis of 10-years survival difference in Sex across different age groups with fine-gray model. S8 Fig. survival difference curves between sex and age group without (A, C) or with (B, D) adjustment. S9 Fig. forest plot for survival difference between sex and age group in cox model. S10 Fig. Survival curves for sex and age groups in Fine-Gray model. S11 Fig. death difference curves between age and sex groups without (A, C) or with (B, D) adjustment. S12 Fig. forest plot for survival difference between age and sex groups in Fine-Gray model. (ZIP) [file pone.0328876.s001.zip › supplementary materials/S5 Figure.tif]

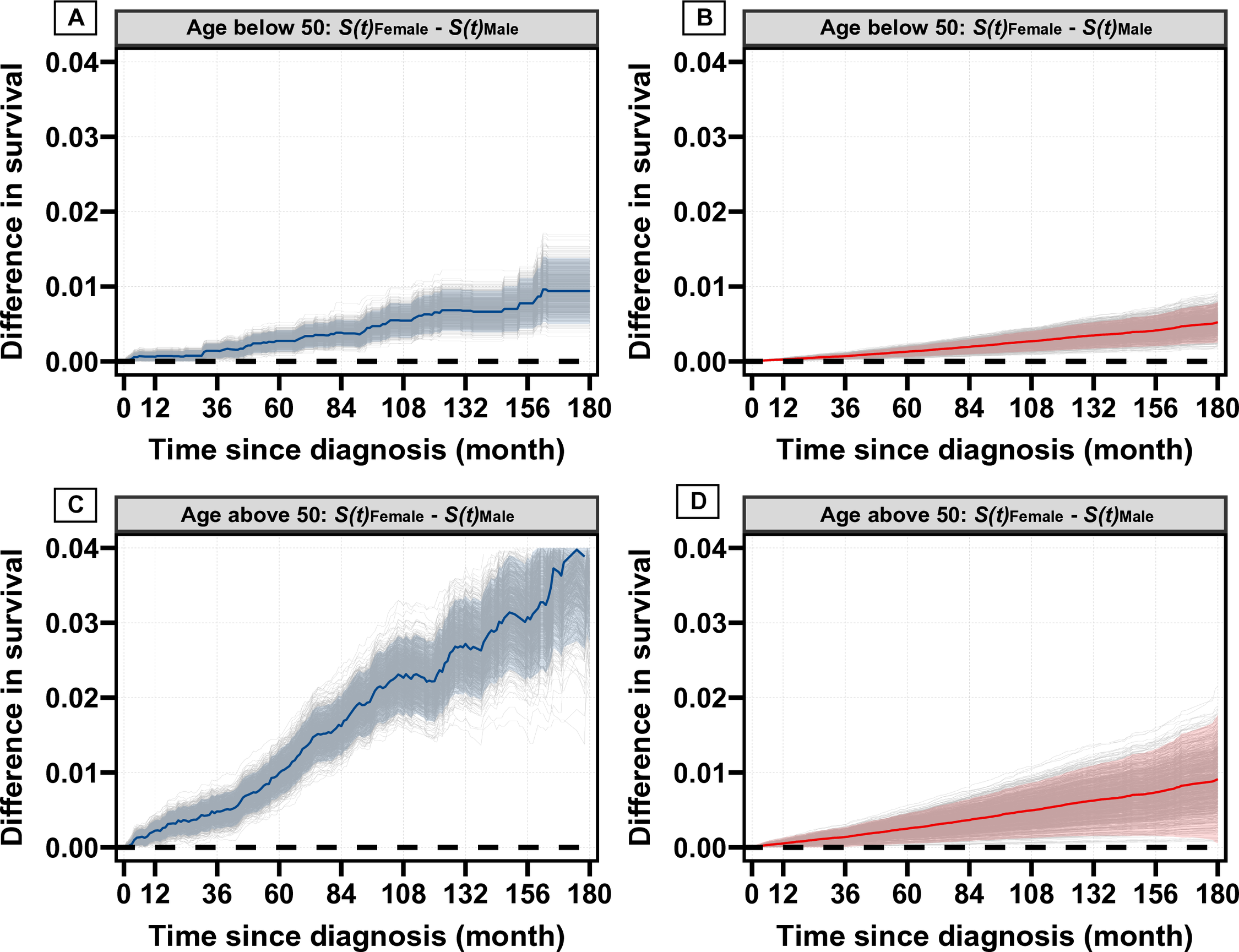

Supplement: S1 File — S1 Text. R codes. S1 Table. Interaction analysis of sex and age in the adjusted Fine-Gray model based on age cutoff of 50. S2 Table. Interaction analysis of sex and age in the adjusted Cox model based on age cutoff of 55. S3 Table. Interaction analysis of sex and age in the adjusted Fine-Gray model based on age cutoff of 55. S1 Fig. Flowchart of patents selection. S2 Fig. Survival curves for Sex in Fine-Gray model. S3 Fig. Death difference curves between sexes in Fine-Gray model without (A) or with (D) adjustment. S4 Fig. subgroup analysis of hazard ratios between sexes across different age groups with cox model. S5 Fig. subgroup analysis of hazard ratios between sexes across different age groups with Fine-Gray model. S6 Fig. subgroup analysis of 10-years survival difference in Sex across different age groups with cox model. S7 Fig. subgroup analysis of 10-years survival difference in Sex across different age groups with fine-gray model. S8 Fig. survival difference curves between sex and age group without (A, C) or with (B, D) adjustment. S9 Fig. forest plot for survival difference between sex and age group in cox model. S10 Fig. Survival curves for sex and age groups in Fine-Gray model. S11 Fig. death difference curves between age and sex groups without (A, C) or with (B, D) adjustment. S12 Fig. forest plot for survival difference between age and sex groups in Fine-Gray model. (ZIP) [file pone.0328876.s001.zip › supplementary materials/S8 Figure.tif]

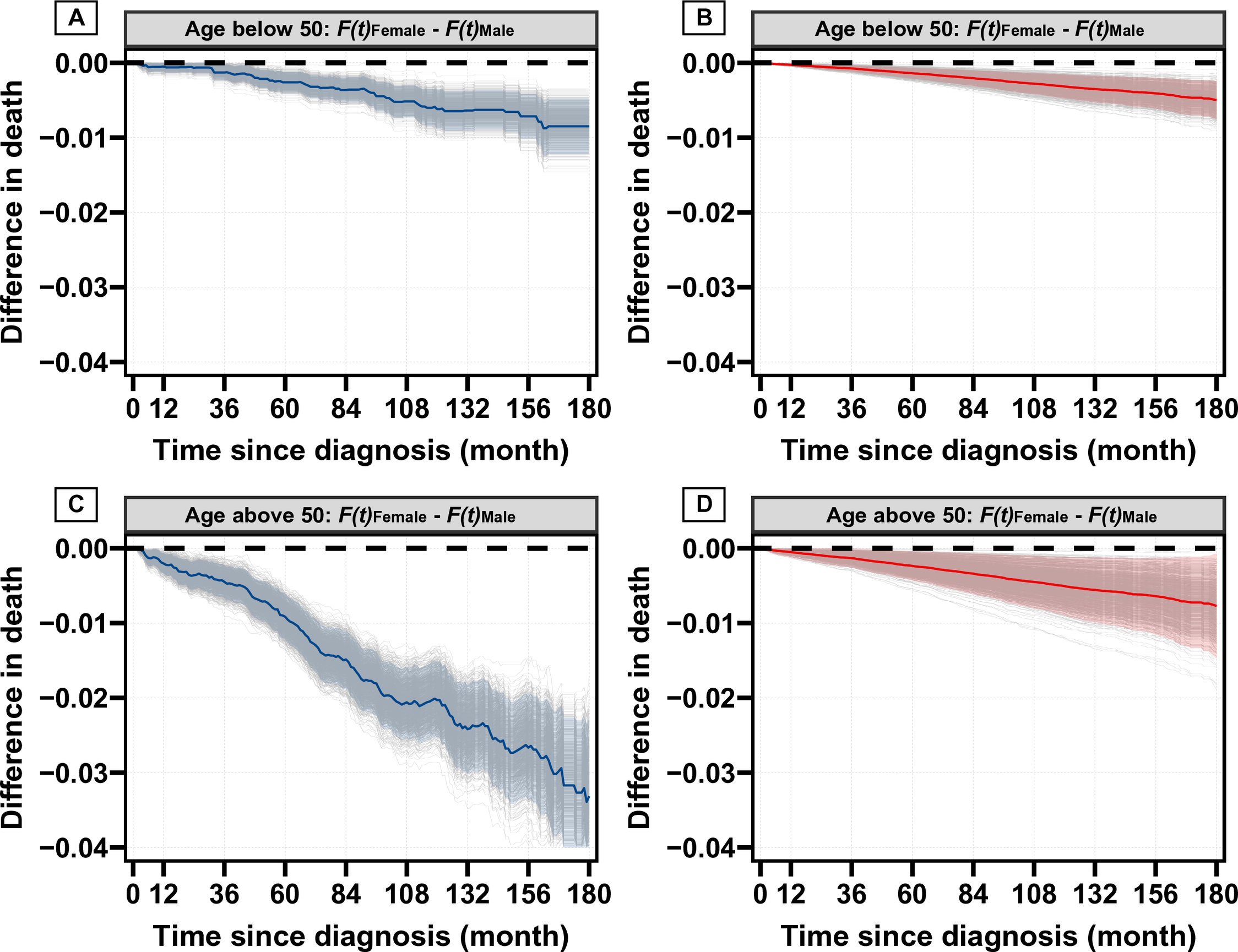

Supplement: S1 File — S1 Text. R codes. S1 Table. Interaction analysis of sex and age in the adjusted Fine-Gray model based on age cutoff of 50. S2 Table. Interaction analysis of sex and age in the adjusted Cox model based on age cutoff of 55. S3 Table. Interaction analysis of sex and age in the adjusted Fine-Gray model based on age cutoff of 55. S1 Fig. Flowchart of patents selection. S2 Fig. Survival curves for Sex in Fine-Gray model. S3 Fig. Death difference curves between sexes in Fine-Gray model without (A) or with (D) adjustment. S4 Fig. subgroup analysis of hazard ratios between sexes across different age groups with cox model. S5 Fig. subgroup analysis of hazard ratios between sexes across different age groups with Fine-Gray model. S6 Fig. subgroup analysis of 10-years survival difference in Sex across different age groups with cox model. S7 Fig. subgroup analysis of 10-years survival difference in Sex across different age groups with fine-gray model. S8 Fig. survival difference curves between sex and age group without (A, C) or with (B, D) adjustment. S9 Fig. forest plot for survival difference between sex and age group in cox model. S10 Fig. Survival curves for sex and age groups in Fine-Gray model. S11 Fig. death difference curves between age and sex groups without (A, C) or with (B, D) adjustment. S12 Fig. forest plot for survival difference between age and sex groups in Fine-Gray model. (ZIP) [file pone.0328876.s001.zip › supplementary materials/S11 Figure.tif]

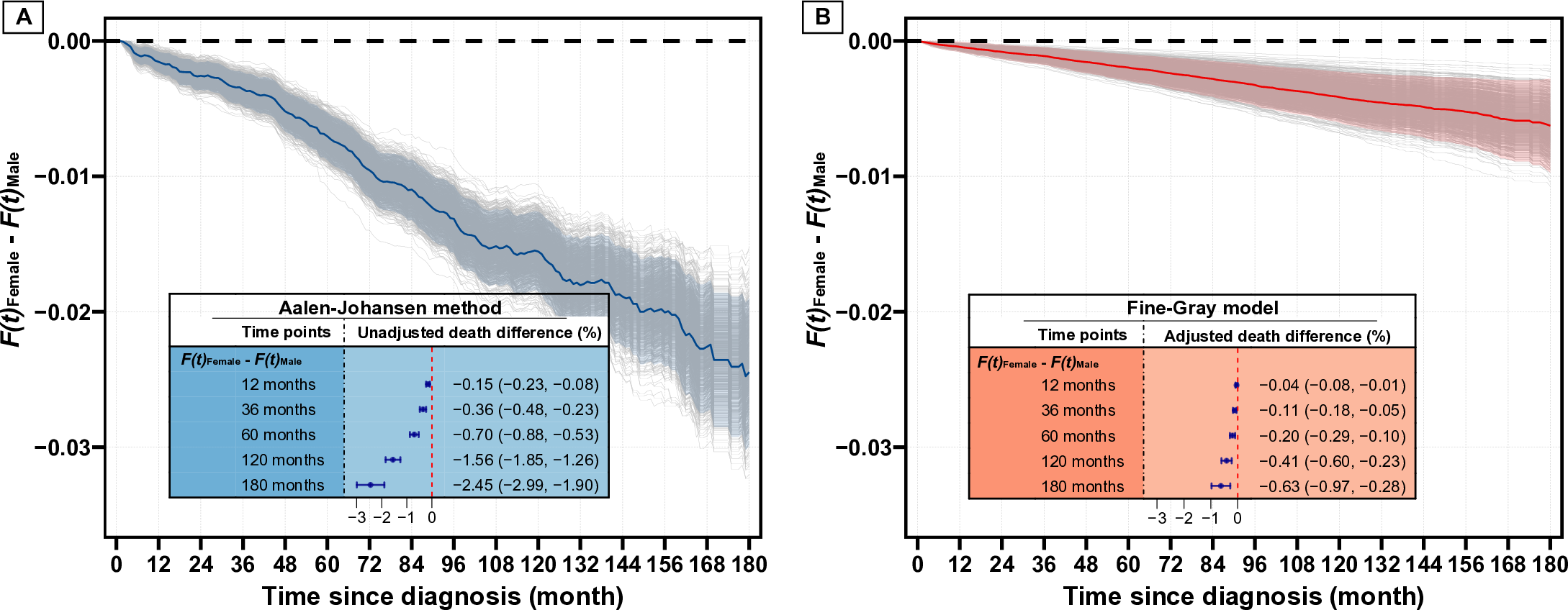

Supplement: S1 File — S1 Text. R codes. S1 Table. Interaction analysis of sex and age in the adjusted Fine-Gray model based on age cutoff of 50. S2 Table. Interaction analysis of sex and age in the adjusted Cox model based on age cutoff of 55. S3 Table. Interaction analysis of sex and age in the adjusted Fine-Gray model based on age cutoff of 55. S1 Fig. Flowchart of patents selection. S2 Fig. Survival curves for Sex in Fine-Gray model. S3 Fig. Death difference curves between sexes in Fine-Gray model without (A) or with (D) adjustment. S4 Fig. subgroup analysis of hazard ratios between sexes across different age groups with cox model. S5 Fig. subgroup analysis of hazard ratios between sexes across different age groups with Fine-Gray model. S6 Fig. subgroup analysis of 10-years survival difference in Sex across different age groups with cox model. S7 Fig. subgroup analysis of 10-years survival difference in Sex across different age groups with fine-gray model. S8 Fig. survival difference curves between sex and age group without (A, C) or with (B, D) adjustment. S9 Fig. forest plot for survival difference between sex and age group in cox model. S10 Fig. Survival curves for sex and age groups in Fine-Gray model. S11 Fig. death difference curves between age and sex groups without (A, C) or with (B, D) adjustment. S12 Fig. forest plot for survival difference between age and sex groups in Fine-Gray model. (ZIP) [file pone.0328876.s001.zip › supplementary materials/S3 Figure.tif]

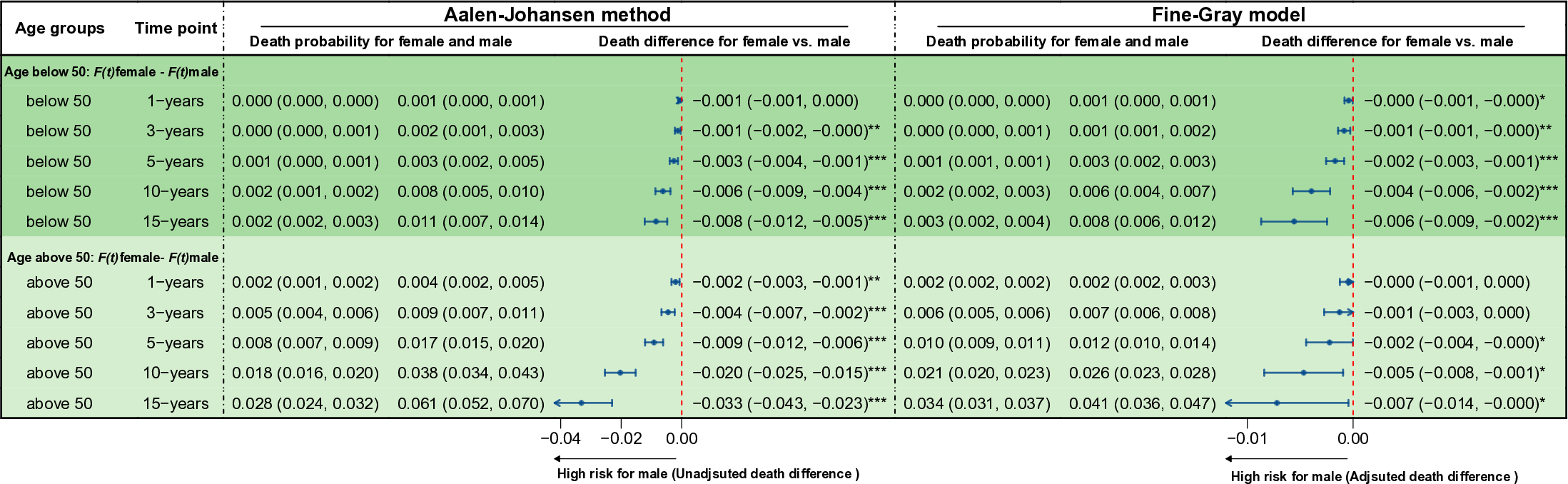

Supplement: S1 File — S1 Text. R codes. S1 Table. Interaction analysis of sex and age in the adjusted Fine-Gray model based on age cutoff of 50. S2 Table. Interaction analysis of sex and age in the adjusted Cox model based on age cutoff of 55. S3 Table. Interaction analysis of sex and age in the adjusted Fine-Gray model based on age cutoff of 55. S1 Fig. Flowchart of patents selection. S2 Fig. Survival curves for Sex in Fine-Gray model. S3 Fig. Death difference curves between sexes in Fine-Gray model without (A) or with (D) adjustment. S4 Fig. subgroup analysis of hazard ratios between sexes across different age groups with cox model. S5 Fig. subgroup analysis of hazard ratios between sexes across different age groups with Fine-Gray model. S6 Fig. subgroup analysis of 10-years survival difference in Sex across different age groups with cox model. S7 Fig. subgroup analysis of 10-years survival difference in Sex across different age groups with fine-gray model. S8 Fig. survival difference curves between sex and age group without (A, C) or with (B, D) adjustment. S9 Fig. forest plot for survival difference between sex and age group in cox model. S10 Fig. Survival curves for sex and age groups in Fine-Gray model. S11 Fig. death difference curves between age and sex groups without (A, C) or with (B, D) adjustment. S12 Fig. forest plot for survival difference between age and sex groups in Fine-Gray model. (ZIP) [file pone.0328876.s001.zip › supplementary materials/S12 Figure.tif]

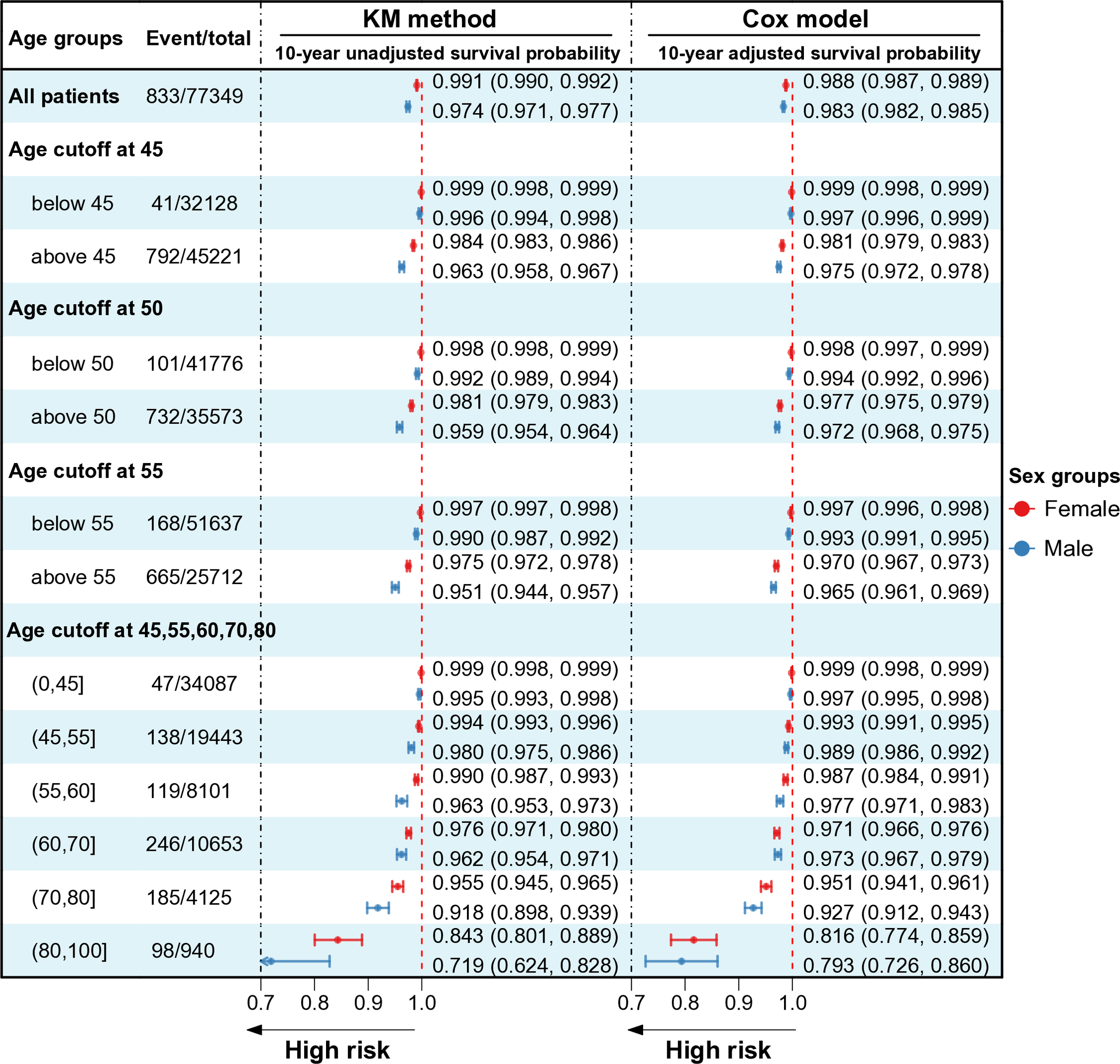

Supplement: S1 File — S1 Text. R codes. S1 Table. Interaction analysis of sex and age in the adjusted Fine-Gray model based on age cutoff of 50. S2 Table. Interaction analysis of sex and age in the adjusted Cox model based on age cutoff of 55. S3 Table. Interaction analysis of sex and age in the adjusted Fine-Gray model based on age cutoff of 55. S1 Fig. Flowchart of patents selection. S2 Fig. Survival curves for Sex in Fine-Gray model. S3 Fig. Death difference curves between sexes in Fine-Gray model without (A) or with (D) adjustment. S4 Fig. subgroup analysis of hazard ratios between sexes across different age groups with cox model. S5 Fig. subgroup analysis of hazard ratios between sexes across different age groups with Fine-Gray model. S6 Fig. subgroup analysis of 10-years survival difference in Sex across different age groups with cox model. S7 Fig. subgroup analysis of 10-years survival difference in Sex across different age groups with fine-gray model. S8 Fig. survival difference curves between sex and age group without (A, C) or with (B, D) adjustment. S9 Fig. forest plot for survival difference between sex and age group in cox model. S10 Fig. Survival curves for sex and age groups in Fine-Gray model. S11 Fig. death difference curves between age and sex groups without (A, C) or with (B, D) adjustment. S12 Fig. forest plot for survival difference between age and sex groups in Fine-Gray model. (ZIP) [file pone.0328876.s001.zip › supplementary materials/S6 Figure.tif]

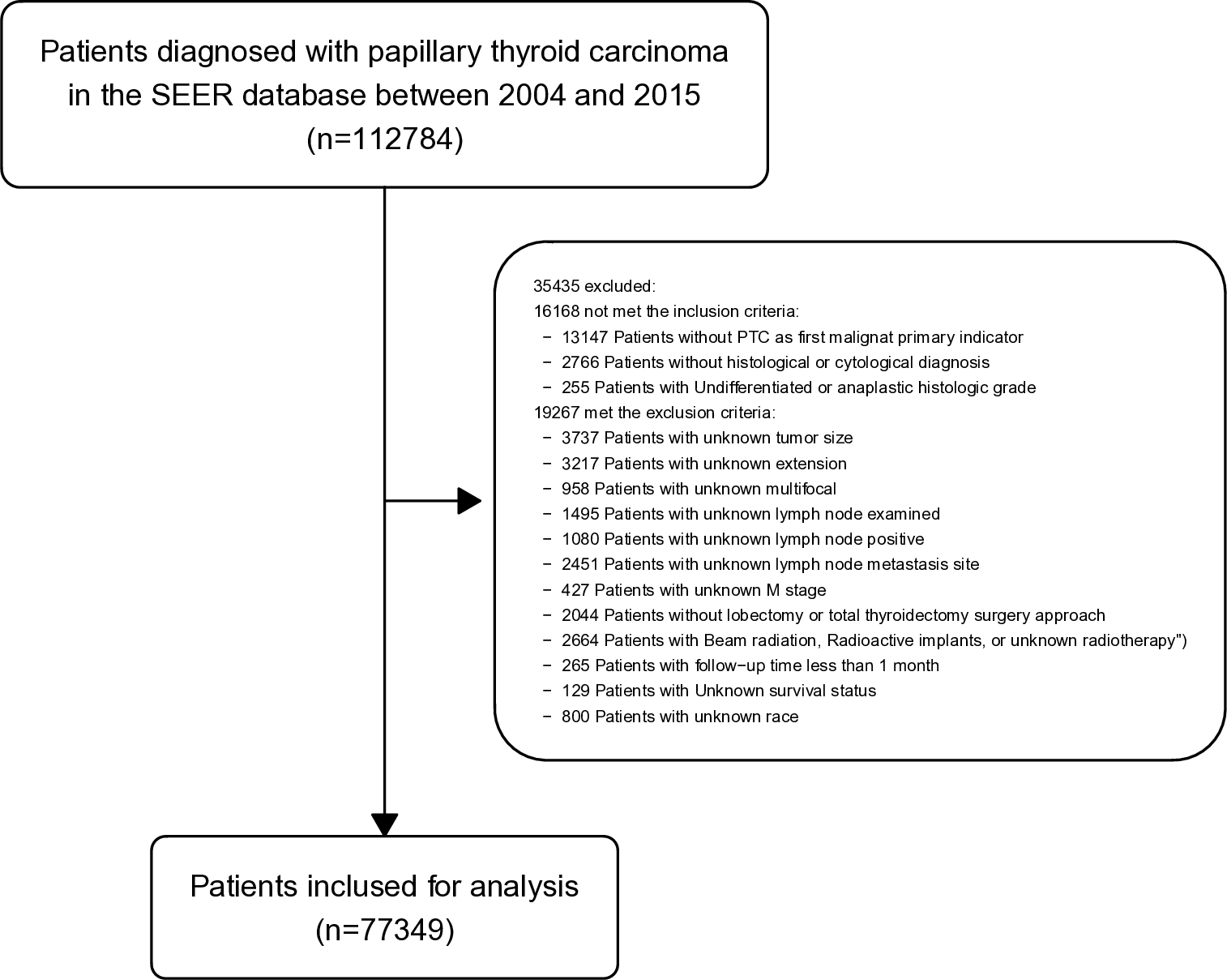

Supplement: S1 File — S1 Text. R codes. S1 Table. Interaction analysis of sex and age in the adjusted Fine-Gray model based on age cutoff of 50. S2 Table. Interaction analysis of sex and age in the adjusted Cox model based on age cutoff of 55. S3 Table. Interaction analysis of sex and age in the adjusted Fine-Gray model based on age cutoff of 55. S1 Fig. Flowchart of patents selection. S2 Fig. Survival curves for Sex in Fine-Gray model. S3 Fig. Death difference curves between sexes in Fine-Gray model without (A) or with (D) adjustment. S4 Fig. subgroup analysis of hazard ratios between sexes across different age groups with cox model. S5 Fig. subgroup analysis of hazard ratios between sexes across different age groups with Fine-Gray model. S6 Fig. subgroup analysis of 10-years survival difference in Sex across different age groups with cox model. S7 Fig. subgroup analysis of 10-years survival difference in Sex across different age groups with fine-gray model. S8 Fig. survival difference curves between sex and age group without (A, C) or with (B, D) adjustment. S9 Fig. forest plot for survival difference between sex and age group in cox model. S10 Fig. Survival curves for sex and age groups in Fine-Gray model. S11 Fig. death difference curves between age and sex groups without (A, C) or with (B, D) adjustment. S12 Fig. forest plot for survival difference between age and sex groups in Fine-Gray model. (ZIP) [file pone.0328876.s001.zip › supplementary materials/S1 Figure.tif]

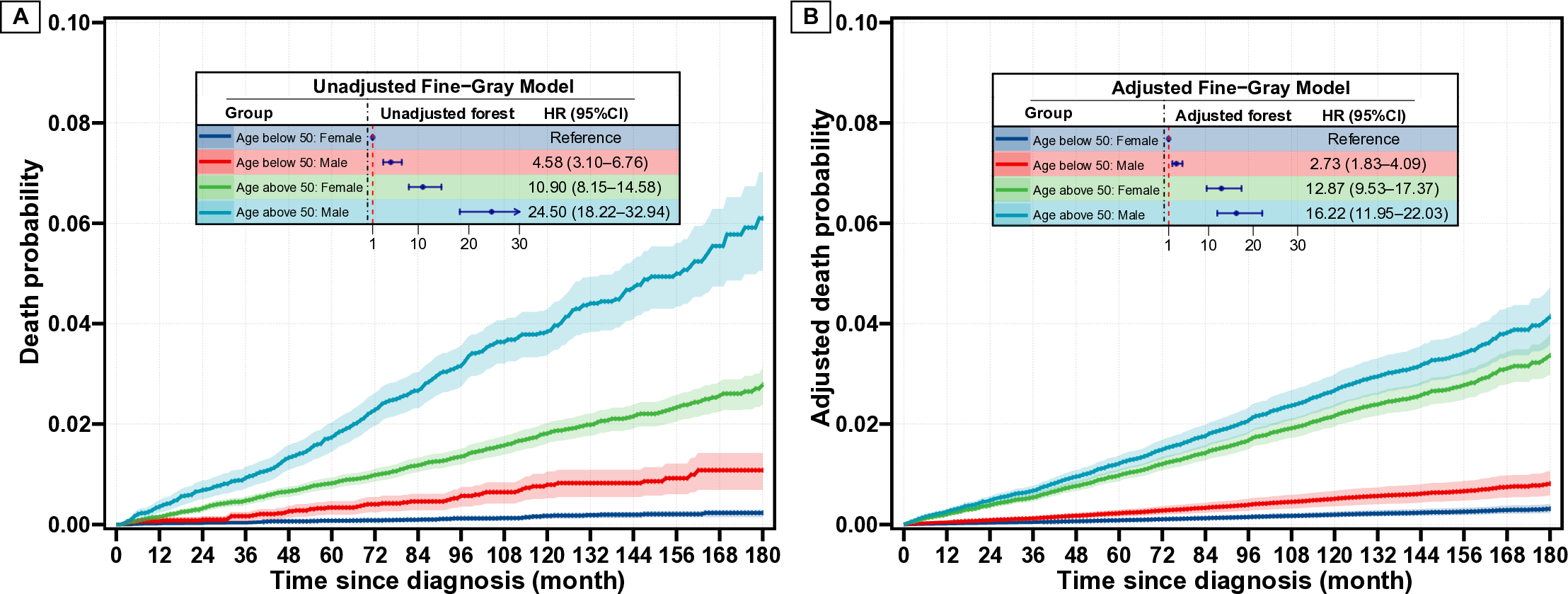

Supplement: S1 File — S1 Text. R codes. S1 Table. Interaction analysis of sex and age in the adjusted Fine-Gray model based on age cutoff of 50. S2 Table. Interaction analysis of sex and age in the adjusted Cox model based on age cutoff of 55. S3 Table. Interaction analysis of sex and age in the adjusted Fine-Gray model based on age cutoff of 55. S1 Fig. Flowchart of patents selection. S2 Fig. Survival curves for Sex in Fine-Gray model. S3 Fig. Death difference curves between sexes in Fine-Gray model without (A) or with (D) adjustment. S4 Fig. subgroup analysis of hazard ratios between sexes across different age groups with cox model. S5 Fig. subgroup analysis of hazard ratios between sexes across different age groups with Fine-Gray model. S6 Fig. subgroup analysis of 10-years survival difference in Sex across different age groups with cox model. S7 Fig. subgroup analysis of 10-years survival difference in Sex across different age groups with fine-gray model. S8 Fig. survival difference curves between sex and age group without (A, C) or with (B, D) adjustment. S9 Fig. forest plot for survival difference between sex and age group in cox model. S10 Fig. Survival curves for sex and age groups in Fine-Gray model. S11 Fig. death difference curves between age and sex groups without (A, C) or with (B, D) adjustment. S12 Fig. forest plot for survival difference between age and sex groups in Fine-Gray model. (ZIP) [file pone.0328876.s001.zip › supplementary materials/S10 Figure.tif]

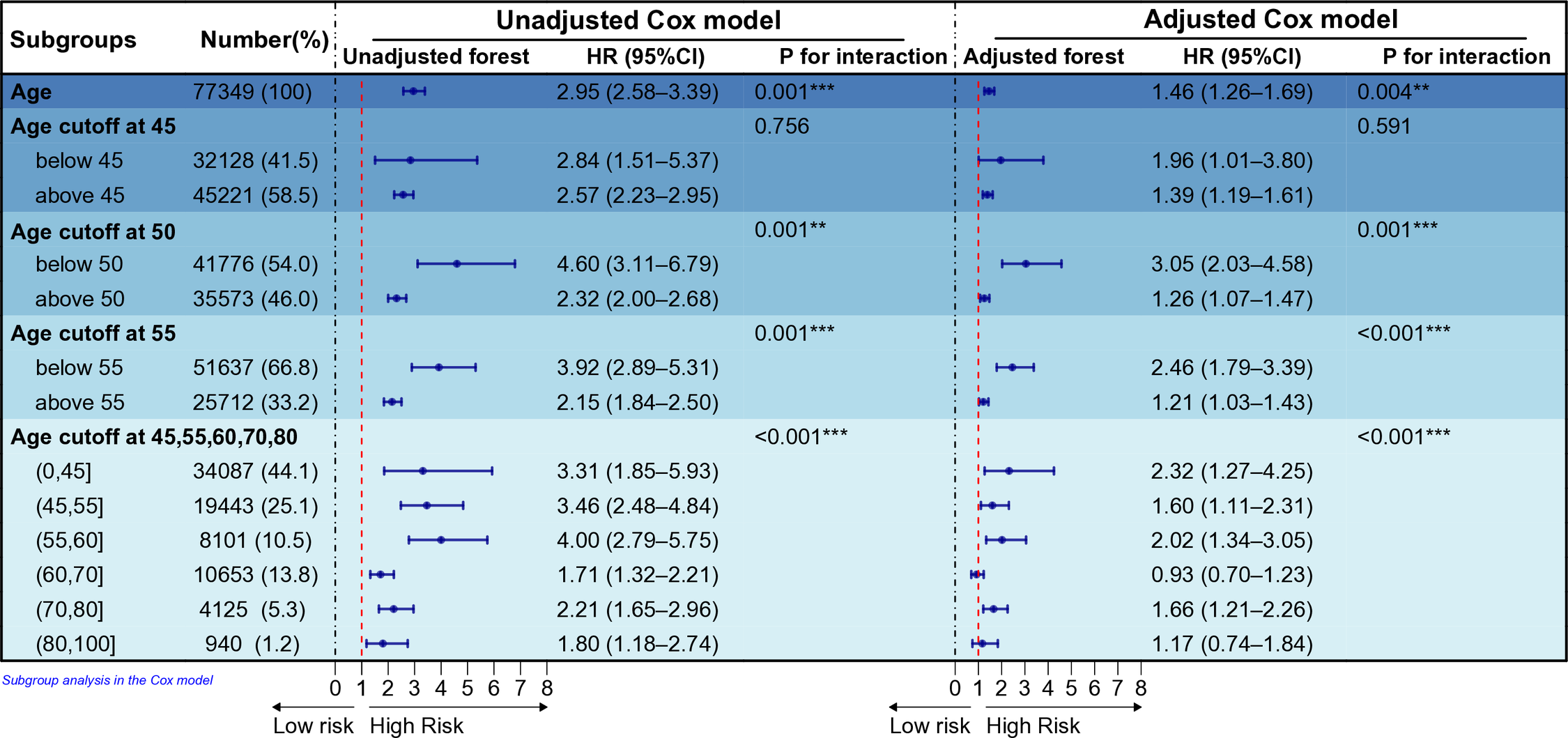

Supplement: S1 File — S1 Text. R codes. S1 Table. Interaction analysis of sex and age in the adjusted Fine-Gray model based on age cutoff of 50. S2 Table. Interaction analysis of sex and age in the adjusted Cox model based on age cutoff of 55. S3 Table. Interaction analysis of sex and age in the adjusted Fine-Gray model based on age cutoff of 55. S1 Fig. Flowchart of patents selection. S2 Fig. Survival curves for Sex in Fine-Gray model. S3 Fig. Death difference curves between sexes in Fine-Gray model without (A) or with (D) adjustment. S4 Fig. subgroup analysis of hazard ratios between sexes across different age groups with cox model. S5 Fig. subgroup analysis of hazard ratios between sexes across different age groups with Fine-Gray model. S6 Fig. subgroup analysis of 10-years survival difference in Sex across different age groups with cox model. S7 Fig. subgroup analysis of 10-years survival difference in Sex across different age groups with fine-gray model. S8 Fig. survival difference curves between sex and age group without (A, C) or with (B, D) adjustment. S9 Fig. forest plot for survival difference between sex and age group in cox model. S10 Fig. Survival curves for sex and age groups in Fine-Gray model. S11 Fig. death difference curves between age and sex groups without (A, C) or with (B, D) adjustment. S12 Fig. forest plot for survival difference between age and sex groups in Fine-Gray model. (ZIP) [file pone.0328876.s001.zip › supplementary materials/S4 Figure.tif]

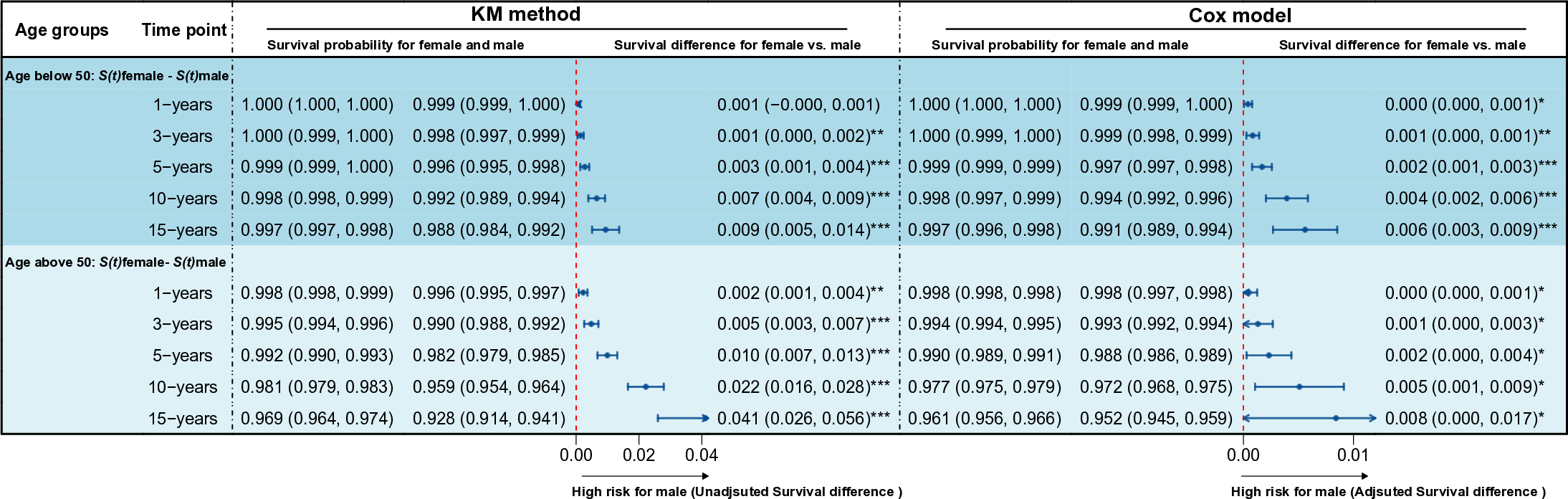

Supplement: S1 File — S1 Text. R codes. S1 Table. Interaction analysis of sex and age in the adjusted Fine-Gray model based on age cutoff of 50. S2 Table. Interaction analysis of sex and age in the adjusted Cox model based on age cutoff of 55. S3 Table. Interaction analysis of sex and age in the adjusted Fine-Gray model based on age cutoff of 55. S1 Fig. Flowchart of patents selection. S2 Fig. Survival curves for Sex in Fine-Gray model. S3 Fig. Death difference curves between sexes in Fine-Gray model without (A) or with (D) adjustment. S4 Fig. subgroup analysis of hazard ratios between sexes across different age groups with cox model. S5 Fig. subgroup analysis of hazard ratios between sexes across different age groups with Fine-Gray model. S6 Fig. subgroup analysis of 10-years survival difference in Sex across different age groups with cox model. S7 Fig. subgroup analysis of 10-years survival difference in Sex across different age groups with fine-gray model. S8 Fig. survival difference curves between sex and age group without (A, C) or with (B, D) adjustment. S9 Fig. forest plot for survival difference between sex and age group in cox model. S10 Fig. Survival curves for sex and age groups in Fine-Gray model. S11 Fig. death difference curves between age and sex groups without (A, C) or with (B, D) adjustment. S12 Fig. forest plot for survival difference between age and sex groups in Fine-Gray model. (ZIP) [file pone.0328876.s001.zip › supplementary materials/S9 Figure.tif]
